# Pathogenic Mechanisms of Two Novel Variants in the GJB1 Gene

**DOI:** 10.1155/humu/7388252

**Published:** 2026-10-09

**Authors:** Fenliang Xiao, Miaojing Xu, Wei Li, Zijian Xu, Haishan Jiang, Shaogang Qu

**Affiliations:** ^1^ Department of Neurology Nanfang Hospital Southern Medical University Guangzhou Guangdong China fimmu.com; ^2^ Key Laboratory of Mental Health of the Ministry of Education Southern Medical University Guangzhou Guangdong China fimmu.com; ^3^ Guangdong-Hong Kong-Macao Greater Bay Area Center for Brain Science and Brain-Inspired Intelligence Guangzhou Guangdong China

**Keywords:** CMT, *GJB1*, *GJB1* c.124A > G, *GJB1* c.130dupT, novel variant

## Abstract

**Background and Aims:**

Charcot–Marie–Tooth disease X‐linked Type 1 (CMTX1), caused by *GJB1* mutations, presents diagnostic challenges due to phenotypic heterogeneity and novel variants. This study reviews clinical data from 2021–2024 and investigates the mechanisms of two novel *GJB1* variants.

**Methods:**

We retrospectively analyzed eight male patients, evaluating clinical signs, electrophysiology, and sural nerve biopsies. Pathogenicity of novel variants was assessed via bioinformatics, ChimeraX molecular modeling, and HeLa cell models focusing on protein localization and channel function (scratch‐loading/DAPI assays).

**Results:**

Patients showed typical distal muscle atrophy and intermediate conduction velocities; three exhibited rare CNS white matter abnormalities. Two novel variants were identified: c.130dupT and c.124A > G. c.130dupT led to a complete “loss of expression” of the full‐length Cx32 protein. c.124A > G resulted in significant cytoplasmic protein retention (*p* < 0.05). Molecular modeling revealed a physical constriction (1.229 Å) in the channel bottleneck and an increased electrostatic barrier. Functional assays confirmed significant impairment in both gap junction and hemichannel activity (*p* < 0.01).

**Interpretation:**

This study identifies two previously unreported *GJB1* variants and elucidates their distinct pathogenic mechanisms: c.130dupT acts through loss of expression, whereas c.124A > G causes functional defect via structural channel constriction. These findings expand the genetic spectrum of CMTX1 and provide a basis for precise molecular diagnosis.

## 1. Introduction

Charcot–Marie–Tooth disease (CMT) is a group of hereditary peripheral neuropathies classified as rare diseases. Mutations in multiple genes, including *PMP22*, *MPZ*, *MFN*2, and gap junction protein beta‐1 (*GJB1*, OMIM 304040) gene, can cause CMT [1]. X‐linked dominant Charcot–Marie–Tooth disease Type 1 (CMTX1, OMIM 302800) is the second most common subtype of CMT, accounting for approximately 13.5%–21.9% of cases [2, 3]. Participants typically exhibit intermediate NCV abnormalities. The causative gene, *GJB1*, located on the X chromosome, encodes Connexin32 (Cx32), a member of the connexin family responsible for forming gap junction channels (GJCs) between adjacent cells [4]. Cx32 is primarily expressed in Schwann cells, especially in the noncompact regions of myelin. It is a tetraspanin protein consisting of four transmembrane domains (TM1, TM2, TM3, and TM4), one cytoplasmic loop (CL), two extracellular loops (EC1 and EC2), as well as intracellular N‐terminus and C‐terminus [5, 6]. Six Cx32 monomers assemble into a hexameric hemichannel, and docking of two hemichannels from adjacent cells forms a GJC. [7] These channels permit diffusion of small molecules (< 1.8 kDa), including ions, second messengers, metabolites, and peptides [8]. Dysfunction of GJCs due to Cx32 deficiency or structural abnormalities is a major cause of demyelinating neuropathy [9].

To date, advances in sequencing technologies such as whole exome sequencing, whole genome sequencing, mitochondrial genome sequencing, and transcriptome sequencing have significantly improved diagnostic yield in CMT [10]. However, many novel variants of uncertain significance have been identified, and establishing their pathogenicity remains a critical challenge for accurate diagnosis.

In this study, we retrospectively analyzed the clinical features of eight participants, two of whom carried novel *GJB1* variants. Using bioinformatics and molecular biology approaches, we examined the effects of these *GJB1* variants. Functional experiments further assessed the activity of mutant Cx32, providing evidence that two novel *GJB1* variants should be upgraded to pathogenic status in CMT. We combined the description of the patient′s clinical phenotype with structural studies and molecular and functional analyses of the mutant Cx32 protein to gain new insights into the pathophysiological mechanisms underlying CMT.

## 2. Materials and Methods

### 2.1. Ethical Approval and Clinical Data Collection

This study included participants clinically diagnosed with CMT at Nanfang Hospital between 2021 and 2024. The diagnosis was confirmed independently by two neurologists. Clinical data were collected, including demographic information (sex, age, and family history), clinical manifestations, genetic analysis, electromyography (EMG), brain MRI, and neuropathological findings. Written informed consent for genetic testing and pathological biopsy was obtained from participants or their legal guardians. The study was approved by the Ethics Review Committee of Southern Hospital (NFEC‐2024‐635).

### 2.2. Protein Monomer Modeling and Bioinformatics Analysis

To investigate the impact of mutation on the structure of the *GJB1* (Cx32) protein, comparative protein modeling was performed in this study. The structures of both the wild‐type (WT) (Cx32^WT^) and variant (Cx32^p.S42G^ or Cx32^p.W44Lfs^ ^∗^^3^) proteins were constructed using the Swiss‐Model platform [11] (http://swissmodel. http://expasy.org/). The high‐confidence AlphaFold prediction model (P08033.1.A, GMQE = 0.80) was selected as the template. Visualization, superimposition, and interaction analysis of all structures were conducted using UCSF Chimera X [12]. Backbone displacement was quantitatively assessed by calculating the root‐mean‐square deviation (RMSD) of alpha‐carbon (*Cα*) atoms. Hydrogen bond (H‐bond) analysis was performed at residue position 42, with the restrict parameter applied to ensure quantification of interactions solely within the model. Furthermore, changes in the local microenvironment were evaluated by monitoring alterations in atom counts and space‐filling characteristics.

### 2.3. Data Analysis

Novel mutation sites were compared against normal control populations using the gnomAD public databases. Pathogenicity prediction was performed using SIFT [13] (https://sift. bii. http://a-star.edu.sg/), POLYPHEN [14] (http://genetics. bwh. harvard.edu/pph2/), PROVEAN [15] (http://provean. http://jcvi.org/index. php), and Mutation taster [16] (https://http://www.mutationtaster.org). The pathogenicity of novel mutation sites was evaluated according to the ACMG guidelines.

### 2.4. Cell Culture, Transfection, and Stable Cell Line Generation

The plasmid uses pCMV as a vector, inserted with *GJB1* cDNA fragments carrying the c.130dupT or c.124A > G mutation. For the c.130dupT plasmid, a FLAG tag was introduced downstream of the *GJB1* sequence to assess whether the mutation results in truncated protein production. For the c.124A > G plasmid, an eYFP gene was fused to the C‐terminus of *GJB1* to generate a fluorescent fusion protein (Cx32‐eYFP; 59 kDa), facilitating the selection of stable cell lines and visualization of subcellular localization.

HeLa cells were cultured in Dulbecco′s Modified Eagle Medium supplemented with 10% fetal bovine serum and 1% penicillin–streptomycin–amphotericin B solution. Plasmid transfection was conducted using Lipo6000 (#C0526, Beyotime, China), strictly following the manufacturer′s recommended protocol. Stable expression was achieved through selection with 1 mg/mL G418 (#ST081, Beyotime, China). The selection medium containing G418 was replaced every 2–3 days, and the cells were cultured for 10–14 days. During this period, cells that failed to be transfected with the plasmid gradually died, whereas cells that were successfully transfected and stably expressed the target gene survived and formed monoclonal colonies. The monoclonal colonies were picked and transferred to a 96‐well plate for further culture. After the cells reached confluence, the expression of *GJB1* protein was detected by western blotting (WB) to confirm the establishment of stably transfected cell lines.

### 2.5. Real‐Time PCR

Total RNA was extracted from cells 48 h after transfection, and cDNA was synthesized according to the manufacturer′s instructions (#R323‐01, Vazyme, China). Quantitative real‐time PCR (qRT‐PCR) was performed using qPCR reagents (#Q712‐02, Vazyme, China). GAPDH or *β*‐actin was used as an internal control. Relative gene expression levels were calculated using the 2^−*ΔΔ*Ct^ method. The primer sequences are GJB1 c.124A > G F‐CTGCTCTACCCTGGCTATGC, R‐ CAGGCCGAGCAGCGGTCGCTCTT; GJB1 c.130dupT F‐AACTGGACAGGTTTGTACACC, R‐ACCACCAGCACCATGATTC; GAPDH F‐GGAGCGAGATCCCTCCAAAAT, R‐GGCTGTTGTCATACTTCTCATGG; and *β*‐actin F‐GTCATTCCAAATATGAGATGCGTTG, R‐TGTGGACTTGGGAGAGGACT.

### 2.6. Immunohistochemistry

Participant nerve biopsy tissues were embedded in OCT and sections 5 *μ*m thick by Leica CM1950 cryostat. The sections were fixed in acetone, followed by 3% H_2_O_2_ inactivation of endogenous peroxidase, and then incubated with a primary antibody against Cx32 (#ab300401, Abcam, United Kingdom) at 37°C for 2 h and with a secondary antibody at 37°C for 20 min. Staining was visualized using DAB reagent (3 min), followed by hematoxylin counterstaining (40 s). Images were captured under a Leica bright‐field microscope.

### 2.7. Immunofluorescence (IF)

Transfected cells on slides in 24‐well plates were fixed in methanol at −20°C and blocked with 5% bovine serum albumin. Then, they incubated with anti‐Cx32 primary antibody (1:200 dilution) at 4°C overnight, followed by secondary antibody incubation at room temperature for 1 h. Nuclei were stained with 4 ^′^,6‐diamidino‐2‐phenylindole (DAPI) for 5 min. Fluorescence images were acquired using a Nikon or Olympus IX73 microscope. The mean fluorescence intensity of Cx32 was quantified using ImageJ (NIH) software. Three independent experiments were conducted, with five random fields analyzed per experiment.

### 2.8. WB

Protein extraction was performed using a cell‐surface biotinylation method for membrane protein enrichment, as previously described. At 24 h after transfection, cell surface proteins were labeled with sulfo‐NHS‐SS‐biotin (#21331, Thermo Fisher, United States; 0.5 mg/mL, pH 8.0) for 20 min on ice, quenched with 100 mM glycine, and lysed. Lysates were centrifuged, and supernatants were collected. Biotinylated proteins were captured with Pierce Streptavidin Agarose (#20347, Thermo Fisher, United States). Membrane and cytoplasmic fractions were separated for WB.

Proteins were resolved on 10% or 7.5% SDS‐PAGE gels (the latter for membrane proteins), transferred to nitrocellulose membranes, and probed with primary antibodies (Cx32, #PA1472S, Abmart, China, 1:500; *β*‐actin, #20536‐1‐AP, Proteintech, China, 1:500; ATP1A1, #55187‐1‐AP, Proteintech, 1:500). After overnight incubation at 4°C, membranes were washed and incubated with HRP‐conjugated secondary antibody (#A0208, Beyotime, China; 1:1000) for 1 h at room temperature. Signals were detected with an ECL kit (#1705061, Bio‐Rad, United States), and band intensity was quantified with ImageJ2 (Version 2.14.0).

### 2.9. Bioinformatic Analysis of Hexameric Structure

Models of the WT (Cx32^WT^) and mutant (Cx32^p.S42G^) proteins were constructed using Swiss‐Model based on the 9qnf template. The models were subjected to energy minimization using Chimera X (v1.11.1) and its built‐in tools. The mutant and WT models were aligned using the “mm” command, and the RMSD of backbone atoms was calculated to assess the impact of the mutation on overall protein folding. Hydrogen bond distribution at the subunit interface was detected separately for the WT and mutant models using the hbonds command. The key residue at position 42 within the channel was selected, and the distance between the centers of mass of opposing subunits was measured using the distance tool. The electrostatic potential distribution on the protein surface was calculated and rendered using the Coulombic Surface Coloring tool. The potential calculation employed default dielectric constant and atomic charge parameters, with a red (negative)–white (neutral)–blue (positive) color scale to visually represent changes in the electrochemical environment within the pore.

### 2.10. Scrape‐Loading‐Dye Transfer Assay

Gap junctional intercellular communication was assessed using the scrape‐loading‐dye transfer method. Briefly, cells at 95%–100% confluence were scraped in a straight line along the middle of six‐well plates using a sterile surgical blade. Detached cells were gently removed by washing with PBS. Lucifer Yellow (#L0144, Sigma‐Aldrich, United States) and rhodamine‐dextran (#R8881, Sigma‐Aldrich, United States) were then added to the medium, and the plates were incubated at 37°C in a 5% CO_2_ incubator for 30 min. After three washes with PBS, cells were fixed with 4% paraformaldehyde for 10 min. Fluorescence images of the scraped areas were captured using an OLYMPUS IX73 fluorescence microscope. The diffusion distance of fluorescence from the scrape edges was measured using ImageJ software. Each experiment was performed in triplicate.

### 2.11. DAPI Uptake Experiment

The experimental procedures were modified based on the literature. Stably transfected HeLa cells were seeded into 96‐well plates and cultured for 12 h, then washed with an extracellular matrix solution (ECM; 138 mM NaCl, 5 mM KCl, 0.4 mM NaH2PO4, 6 mM D‐glucose, 10 mM HEPES, and 60 mM CaCl_2_) and incubated in ECM for 30 min. Subsequently, DAPI solution (#D9542, Sigma‐Aldrich, United States; 5 mg/mL) diluted in ECM was added to the wells. Fluorescence images were immediately captured using a fluorescence microscope to determine baseline fluorescence intensity. After a 5‐min incubation, images were captured again to measure postuptake fluorescence intensity. The fluorescence intensity of DAPI in each group was analyzed using ImageJ, and the difference between baseline and postuptake intensity was calculated. Three independent experiments were performed.

### 2.12. Statistical Analysis

All statistical analyses were performed using GraphPad Prism (Version 10.2.1). For normally distributed data, unpaired *t*‐tests were used for comparisons between two groups, and one‐way ANOVA was used for multiple group comparisons. A *p* value < 0.05 was considered statistically significant.

## 3. Results

### 3.1. Analysis of the Clinical Presentation Spectrum in Eight CMTX1 Patients

This study included eight unrelated CMTX1 patients from our center between 2021 and 2024. The age at consultation ranged from 16 to 40 years, and the age of symptom onset ranged from 6 to 36 years, with a mean age at first onset of 20.86 ± 10.27 years. Their clinical manifestations were highly consistent with classic Charcot–Marie–Tooth disease (Table S1), including distal lower limb weakness, muscle atrophy, gait abnormalities, and mild to moderate distal upper limb atrophy. The initial symptom in most cases was lower limb weakness. Fifty percent of the patients (Probands 3, 4, 6, and 8) had high‐arched feet. Abnormal cranial MRI findings were present in three patients (37.5%). Notably, Proband 4 exhibited not only peripheral nerve involvement but also nystagmus and abnormal signal in the splenium of the corpus callosum.

The mean motor conduction velocity (MCV) of the median nerve was 32.88 m/s, and the abnormality rate for sensory conduction velocity (SCV) was as high as 100% (Table S2). Nerve biopsy pathology revealed loss of large myelinated fibers and myelin splitting (Table S3). An onion bulb formation was observed in Proband 8. *GJB1* gene variants were identified in all eight patients (see Figure 1a and Table S4). These mutations were distributed across different functional domains, including two intronic variants and six exonic variants (Figure 1b). According to the HGMD pro database, six variants had been previously reported in the literature [17–20], whereas two variants (c.124A > G, c.130dupT) had not been reported.

**Figure 1:**
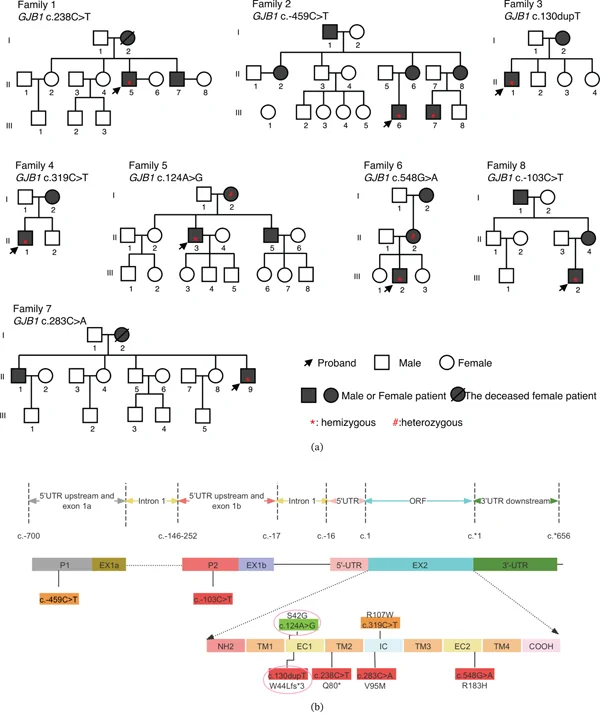
Pedigrees of eight families and schematic representation of GJB1 mutation distribution. (a) Pedigrees are labeled as Family 1–8. Squares represent males, circles represent females, filled symbols represent affected individuals, a circle with a diagonal line represents a deceased female patient, and black arrows indicate the proband of each family. A red asterisk (∗) denotes a hemizygous mutation carrier, and a red hash symbol (#) denotes a heterozygous mutation carrier. (b) Nucleotide numbering for *GJB1* refers to the reference sequence NM_000166.6. The gene structure is depicted as follows: The upper panel illustrates the arrangement of the *GJB1* gene′s untranslated regions (5 ^′^UTR, 3 ^′^UTR), introns, and open reading frame (ORF). P1 and P2 in the middle represent distinct promoter regions; EX1a, EX1b, and EX2 represent the corresponding exons. The lower panel illustrates the corresponding Connexin 32 (Cx32) protein domains: NH2 denotes the amino‐terminus; TM1–4 denote the transmembrane domains, EC1–2 denote the extracellular loops, IC denotes the intracellular loop, and COOH denotes the carboxy‐terminus. Variants distribution: Variants identified in this study are marked with labels of different colors. Variants located in noncoding regions (e.g., c.‐459C > T, c.‐103C > T) are annotated within the promoter/5 ^′^UTR region. Variants located in the coding region (e.g., c.124A > G, c.238C > T, etc.) are annotated below the corresponding protein functional domains, with the amino acid change indicated (e.g., S42G, Q80∗). Pink circles highlight the novel variants sites identified in this study.

### 3.2. Pathogenicity Analysis of Two Novel Mutations

Two novel variants in the *GJB1* gene, c.130dupT (p.Trp44Leufs∗3, p.W44Lfs∗3) and c.124A > G (p.Ser42Gly, p.S42G), were identified in two unrelated families (Family 3 and Family 5, respectively). Sanger sequencing confirmed that both variants cosegregated with the disease phenotype in their respective families. Specifically, in Family 3, the proband′s mother exhibited atrophy of the first dorsal interosseous muscle. Sanger sequencing of the proband′s two elder sisters revealed a WT genotype and a heterozygous c.130dupT mutation, respectively (Figure 2a), indicating that the mother was a heterozygous carrier. Thus, the sequencing results, combined with the mother′s clinical presentation, support cosegregation in this family. In Family 5, both the proband′s mother and younger brother presented with gait abnormalities, with the brother′s symptoms being more severe. Sanger sequencing identified the mother as a heterozygous carrier of the c.124A > G variant (Figure 2b).

**Figure 2:**
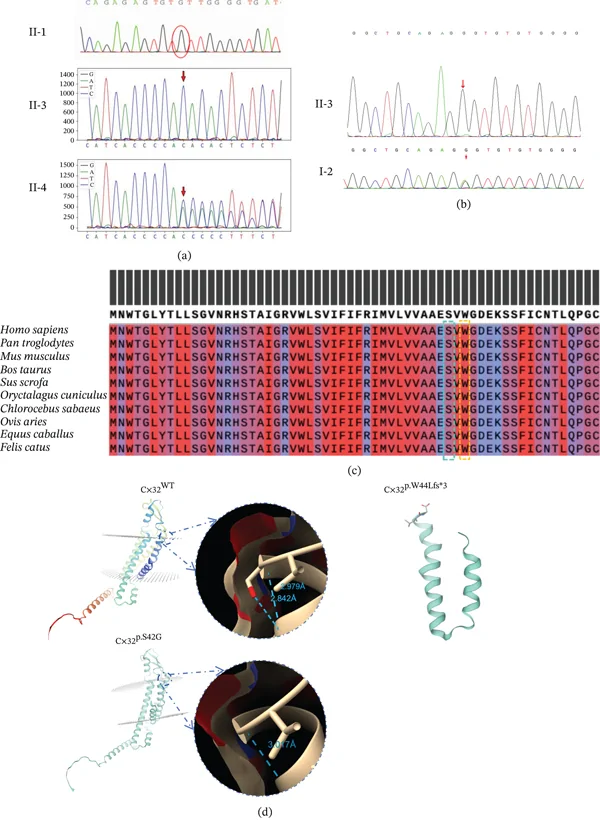
Genetic identification, evolutionary conservation, and structural modeling of the two *GJB1* variants. (a) Sanger sequencing chromatograms for Family 3. The red circle indicates the frameshift mutation c.130dupT in the proband (II‐1). The elder sister (II‐3) is wild‐type, and the younger sister (II‐4) carries the heterozygous variant. (b) Sanger sequencing chromatograms for Family 5. The red arrow indicates the missense mutation c.124A > G. Both the proband (II‐3) and the mother (I‐2) carry this heterozygous variant. (c) Multiple sequence alignment of Cx32 protein across different species. The blue dashed box highlights the highly conserved serine at position 42 (Ser42, S42), and the orange dashed box highlights the highly conserved tryptophan at position 44 (Trp44, W44), suggesting that these residues have profound functional importance during evolution. (d) Swiss‐Model three‐dimensional structural modeling of wild‐type (WT) and mutant Cx32 proteins. The models were constructed based on template P08033.1. The Cx32^p.W44Lfs^ ^∗^^3^ model demonstrates that the mutation leads to severe premature truncation of the protein. The Cx32^p.S42G^ model shows that the mutation does not affect the full‐length protein. The circular inset provides an enlarged view of the hydrogen bond network surrounding residue 42. In the wild‐type model, Ser42 forms two stable hydrogen bonds with lengths of 2.842 and 2.979 Å, respectively. The p.S42G mutation alters the local conformation, resulting in the loss of one hydrogen bond, with the remaining bond distance measured at 3.017 Å.

Neither variant was found in the gnomAD public databases, indicating that they are extremely rare in the general population. Multiple sequence alignment across different species revealed that the Trp44 and Ser42 residues are highly conserved from humans to zebrafish (Figure 2c), suggesting that they play crucial roles in the structural or functional integrity of Cx32. The pathogenicity of these variants was assessed using bioinformatics tools including SIFT, PolyPhen‐2, PROVEAN, and Mutation Assessor (Table 1). The c.124A > G variant was predicted as pathogenic by PolyPhen‐2 (score: 0.987) and MutationTaster (score: 0.99). The c.130dupT variant was also predicted as pathogenic, with an extreme PROVEAN score of −597.540 and a MutationTaster score of 1.0. Using the Swiss‐Model online platform with the P08033.1 monomer as a template, protein monomer models for Cx32^p.S42G^ and Cx32^p.W44Lfs^ ^∗^^3^ were successfully constructed (Figure 2d). The models indicated that the p.S42G missense variant retains the full‐length protein, whereas the p.W44Lfs∗3 frameshift variant severely disrupts the EC1 domain and all downstream key functional structures.

**Table 1:** Pathogenicity classification of novel variants according to ACMG guidelines.

Proband	Variant	PolyPhen2	SIFT	PROVEAN	Mutationtaster	ACMG pathogenic variant classification criteria evidence			Pathogenicity determination
						Strong	Moderate	Support	
3	GJB1 c.130dupT	N/A	N/A	−597.5	1	PVS1	PM2	PP3	P
5	GJB1 c.124A > G	0.987	0.23	−2.274	0.99		PM1 + PM2	PP3 + PP4	LP

*Note:* PolyPhen‐2, score ranges from 0 to 1, score > 0.908 means “Probably damaging.” SIFT, score < 0.05 indicates damaging variant. PROVEAN, score ≤ −2.5 means “damaging,” score > −2.5 means “neutral/tolerated.” Mutationtaster, provides probability of pathogenic variation ranging from 0 to 1; the closer to 1, the higher the pathogenics probability.

Abbreviations: LP, likely pathogenic; P, pathogenic.

Further analysis of the Swiss‐Model predicted structures was performed using Chimera X. Given the significant structural defects evident for the truncating variant, subsequent detailed structural analysis focused solely on the p.S42G variant. Structural superposition of the WT and p.S42G variant monomers revealed a C*α* RMSD of only 0.003 Å, indicating that the variant did not cause severe global misfolding of the protein. However, significant alterations were observed in the local physicochemical environment of the variant site. Quantitative hydrogen‐bond analysis revealed a 50% reduction in intrahelical interactions due to the variant. The WT Ser42 residue maintained two stable hydrogen bonds anchoring the TM1 helix, whereas the variant Gly42 residue retained only one hydrogen bond (distance: 3.017 Å). To further elucidate the physicochemical basis of this structural change, residue properties were analyzed using Project HOPE. The analysis confirmed that the polar serine with a side chain was replaced by a smaller, more flexible, nonpolar glycine. As this site lies at the interface between the TM1 helix and the EC1 domain of Cx32, the reduction in amino acid volume may lead to loss of interactions with the surrounding lipid environment or adjacent residues, such as the loss of hydrogen bonds. Furthermore, the increased local flexibility introduced by glycine may compromise the structural rigidity of the TM1 helix, thereby affecting the stability of Connexin 32 protein and the assembly of GJCs. Bioinformatics prediction yielded a MetaRNN score of 0.819 for this variant, suggesting a high probability of pathogenicity.

Based on the data, the variant generated by *GJB1* c.124A > G is located within a highly conserved region of the Cx32 protein, which constitutes a key functional domain (PM1). Multiple online prediction tools support its pathogenicity, meeting the criteria for PP3 evidence. After excluding population polymorphism (PM2) and integrating clinical familial cosegregation analysis, this variant was preliminarily classified as a likely pathogenic variant according to the ACMG standards. The GJB1 c.130dupT frameshift mutation results in a loss of structure leading to a loss of function (LOF), fulfilling the criteria for PVS1 (very strong evidence of pathogenicity). Both prediction tools indicated pathogenicity, meeting the criteria for PP3 (supporting evidence of pathogenicity). Given its extremely low frequency in the gnomAD public database (PM2), this variant was preliminarily classified as a pathogenic variant according to the ACMG standards (Table 1).

### 3.3. The GJB1 c.130dupT Truncating Mutation Leads to Loss of Full‐Length Cx32 Protein

To investigate the impact of the c.130dupT variant, the expression of Cx32 in patient neural tissue was evaluated. Immunohistochemical staining using an antibody targeting the intracellular domain of Cx32 (residues 96–130) revealed a complete absence of the characteristic brown punctate signal in the patient′s sural nerve compared to the WT control group (Figure 3a), indicating a lack of full‐length Cx32 in vivo. This finding was further validated in a HeLa cell overexpression model. Plasmids were constructed containing the WT *GJB1* coding sequence (CDS) (WT group) or the mutant *GJB1* c.130dupT CDS (c.130dupT group), each fused at the C‐terminus to a 3 × Flag tag sequence, and overexpressed in HeLa cells. qPCR analysis showed no significant difference in *GJB1* mRNA transcript levels between the WT and c.130dupT groups (*p* > 0.05) (Figure 3b), indicating that the c.130dupT mutation did not trigger nonsense‐mediated mRNA decay (NMD). However, neither Cx32 nor the C‐terminal Flag‐tagged protein was detected in the mutant group by WB (Figure 3c) or IF (Figure 3d). Therefore, the mutation resulted in a “loss of expression” of the full‐length Cx32 protein. It results in a complete LOF, consistent with the severe clinical phenotype observed in Family 3.

**Figure 3:**
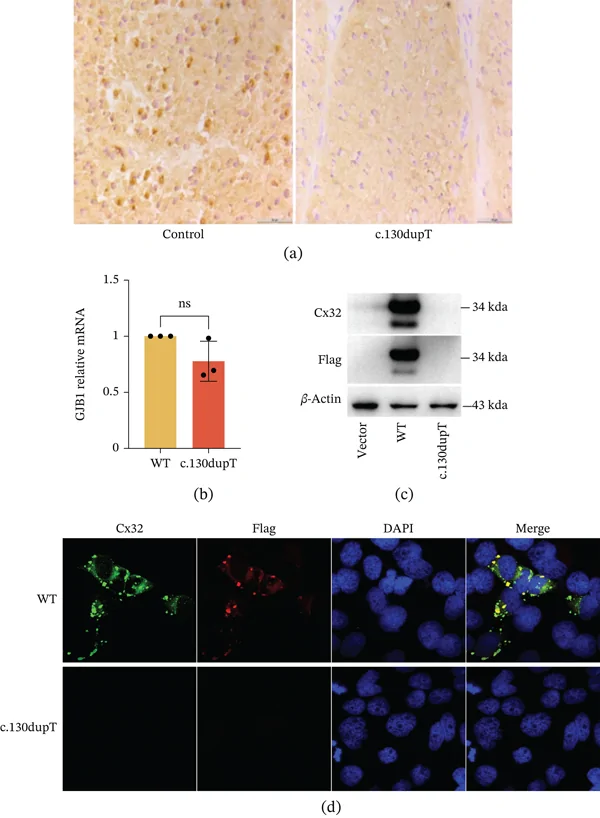
Experimental validation of the impact of the *GJB1* c.130dupT variant on Cx32 expression. (a) Immunohistochemistry (IHC) analysis of Cx32 expression and distribution in nerve tissue from the normal control group (Control) and the proband (c.130dupT). The results show a loss of Cx32‐positive signals in the proband′s tissue compared to the control. Images were captured using a Leica microscope; scale bar = 50 *μ*m. (b) Quantitative real‐time PCR (qPCR) analysis of relative *GJB1* mRNA levels in HeLa cells. Quantitative analysis revealed no statistically significant difference (*ns*) in mRNA transcript levels between the wild‐type (WT) and mutant (c.130dupT) groups. This indicates that this frameshift mutation does not trigger significant nonsense‐mediated mRNA decay (NMD). (c) Western blot (WB) analysis of Cx32 protein overexpression in HeLa cells. Protein expression post‐transfection was detected using anti‐Cx32 and anti‐Flag tag antibodies, with *β*‐actin serving as the loading control. The results show that no distinct protein band was detected in the c.130dupT mutant group compared to the wild type, suggesting that this variant leads to impaired protein synthesis or rapid degradation. (d) Immunofluorescence (IF) analysis of Cx32 protein overexpression in HeLa cells. Green fluorescence represents Cx32, red fluorescence represents the Flag tag, and blue indicates DAPI‐stained nuclei. The images further confirm that almost no protein signal was observed in the c.130dupT mutant group, consistent with the WB results. Images were captured using a Nikon microscope at 40 × magnification.

### 3.4. Conformational and Functional Alterations of the Channel Caused by the *GJB1* c.124A > G Missense Mutation

We performed IF detection of Cx32 protein on patient nerve tissue harboring the c.124A > G mutation. In contrast to the results for the c.130dupT mutation, Cx32 fluorescence was detectable in the nerve, and its level did not differ significantly from that of the control group (Figure 4a). It indicates that the mutation did not affect the full‐length expression of the Cx32 protein. To further investigate the impact of the mutation on Cx32′s biological function, plasmids were similarly constructed and overexpressed in HeLa cells to establish a cellular model, and we conducted a multidimensional evaluation at the transcriptional, translational, and subcellular localization levels.

**Figure 4:**
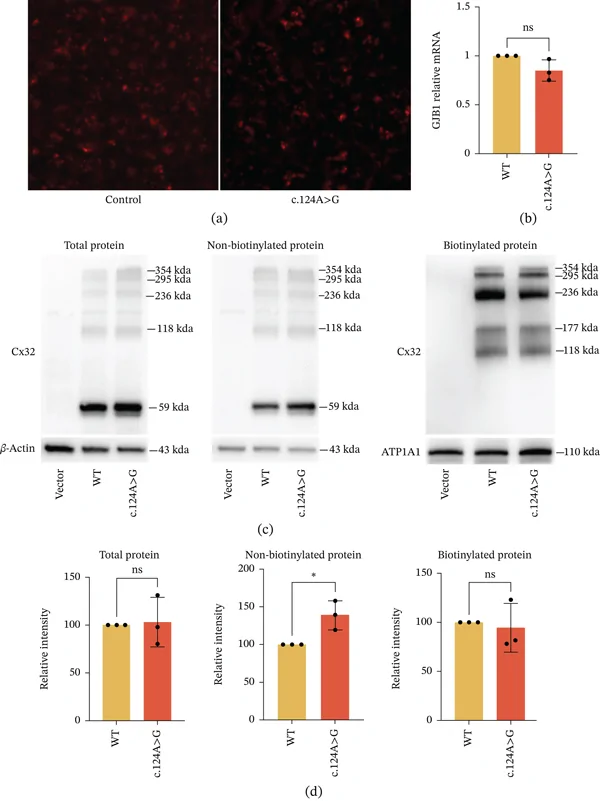
Experimental validation of the effect of the *GJB1* c.124A > G variant on Cx32 expression. (a) Cx32 levels in the proband′s nerve tissue were detected by IHC. No significant difference in Cx32 expression was observed between the normal control group (Control) and the proband′s nerve tissue (c.124A > G). (b) The relative mRNA expression levels of wild‐type (WT) and c.124A > G mutant *GJB1* were detected by qPCR, showing no significant difference (*ns*). (c) Representative blot from the cell surface biotinylation assay. Anti‐Cx32 antibody was used to detect total protein, nonbiotinylated protein (representing cytoplasmic protein), and biotinylated protein (representing membrane surface protein). *β*‐Actin and ATP1A1 served as loading controls for the cytoplasmic and membrane fractions, respectively. Higher molecular weight bands represent the formation of Cx32 multimers. Total and cytoplasmic proteins were separated on a 10% gel, whereas membrane proteins were separated on a 7.5% gel. (d) Quantitative statistical analysis of the protein band intensities from panel (c). Data are presented as the mean ± standard deviation from three independent experiments (*n* = 3). ∗*p* < 0.05; *ns*, not statistically significant.

qPCR results revealed no significant difference in mRNA transcription levels between the c.124A > G mutant and the WT (Figure 4b). Regarding protein expression, cell surface biotinylation assay results (Figure 4c–d) showed no statistically significant differences in the levels of total protein or biotinylated protein (membrane protein) between the WT and mutant groups. However, the level of nonbiotinylated protein (cytoplasmic protein) in the mutant group was significantly higher than in the WT (*p* < 0.05). It suggests that the mutation leads to partial accumulation of the protein in the cytoplasm. Although the amount of protein on the membrane did not reach statistical significance, the distribution ratio of the protein has already been altered.

IF staining results (Figure 5) showed that the WT Cx32 protein was primarily localized to the cell periphery and exhibited good colocalization with the plasma membrane marker ATP1A1. Plot Profile analysis of fluorescence intensity distribution further confirmed that the signal peaks of eYFP (green) and ATP1A1 (red) were highly coincident spatially in the WT group. In the c.124A > G mutant group, partial protein had been transported to the plasma membrane, whereas some aggregated protein granules were also present in the cytoplasm. Plot Profile analysis revealed multiple peaks of green fluorescence intensity in the cytoplasm of the mutant group, whereas coincident peaks at the membrane were attenuated compared to the WT group. This observation is consistent with the results of the cell‐surface biotinylation assay, which collectively indicate that the p.S42G mutation may lead to partial cytoplasmic retention of the Cx32 protein.

**Figure 5:**
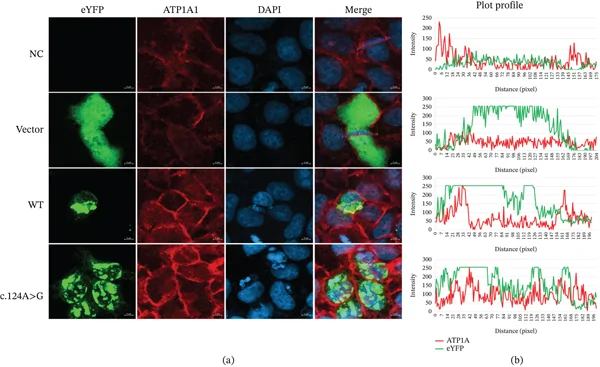
Immunofluorescence localization and colocalization analysis of Cx32 wild‐type and mutant proteins. (a) Representative immunofluorescence images observed in multiple fields of view from three independent experiments. Green indicates eYFP‐tagged Cx32 protein, red indicates the plasma membrane marker ATP1A1, and blue indicates the nuclear marker DAPI. NC denotes the negative control, and Vector denotes the empty vector control. Images were captured using a Zeiss 63x confocal microscope. (b) Plot Profile analysis of fluorescence intensity. The red and green lines represent the fluorescence intensity distribution of ATP1A1 and eYFP, respectively. Colocalization is indicated when both fluorescence intensities are elevated at the same position. The measurement position corresponds to the wavy line in the Merge image.

To further investigate whether the successfully membrane‐inserted protein hexamer undergoes structural or functional alterations, we performed structural analyses of the WT Cx32 (Cx32^WT^) and mutant Cx32 (Cx32^p.S42G^) models constructed based on the 9qnf template in Chimera X. Superimposition of the Cx32^WT^ and Cx32^p.S42G^ models revealed a RMSD of backbone atoms (*Cα*) of only 0.284 Å. This indicates that the single‐point mutation does not disrupt the typical hexameric topology of connexin (Figure 6a), and the mutational effect is primarily localized. However, hydrogen bond analysis showed that the WT subunit possesses 20 hydrogen bonds, whereas the mutant exhibits only 19. The loss of 5% of interfacial interactions suggests that the mutation may weaken the stability of hexamer assembly. Measurement at the key pore‐lining residue position 42 revealed that the *Cα*–*Cα* distance decreased from 21.488 Å in the Cx32^WT^ to 20.259 Å in the Cx32^p.S42G^ (Figure 6b). This physical contraction of 1.229 Å demonstrates a collapse in the channel “bottleneck” region, likely attributable to the smaller side chain of glycine being unable to maintain the original spatial support provided by serine. Coulombic surface analysis indicated that the mutant exhibits a higher local positive electrostatic potential (maximum: Cx32^p.S42G^ 14.24 vs. Cx32^WT^ 13.85 kcal/(mol·e)). The physical constriction of the pore diameter, combined with the altered local electrostatic density, elevates the electrostatic energy barrier for ion traversal of the channel, potentially reducing channel permeability.

**Figure 6:**
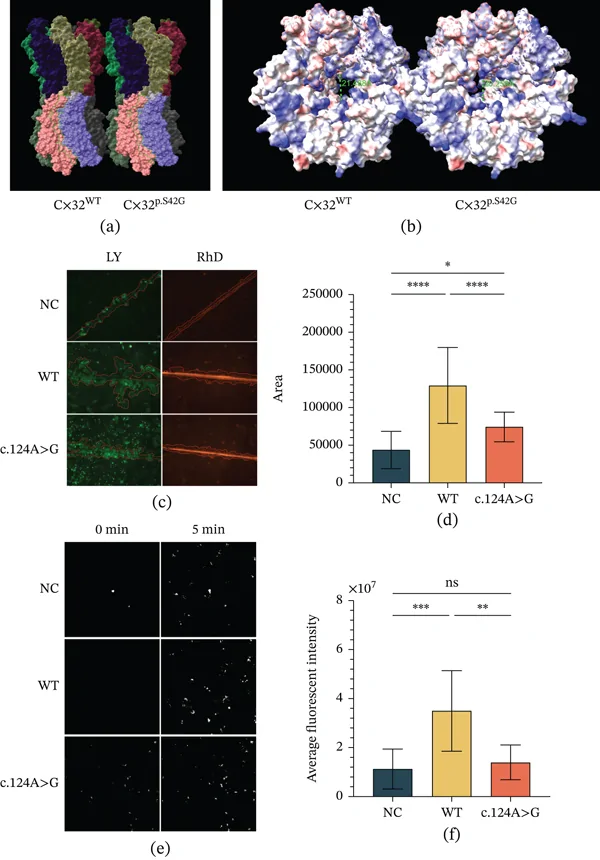
Analysis of hexameric structure and experimental validation of channel function. (a) Overall conformational model of the Cx32 hexamer constructed via homology modeling based on the 9qnf template. The left panel shows the wild‐type (Cx32^WT^), and the right panel shows the mutant (Cx32^p.S42G^). The backbones of the two structures are highly consistent (RMSD 0.284 Å). Different subunits are rendered in different colors. (b) Top view along the pore axis showing the surface electrostatic potential (Coulombic potential) distribution. Red indicates negative potential, and blue indicates positive potential. The green dashed line marks the central span between the C*α* atoms of residue 42. Measurements indicate that the Cx32^p.S42G^ physically constricts the central pore span from 21.488 to 20.259 Å and alters the local positive potential maximum (max: 14.24 vs. 13.85 kcal/(mol·e)), suggesting an increased energy barrier for ion permeation. (c) Gap junction channel function assessed by scrape‐loading dye transfer assay. The red outline demarcates the area of Lucifer Yellow (LY) diffusion from the initial scrape line; the right panel shows the area of Rhodamine (RhD) diffusion. (d) Quantitative analysis of the dye diffusion area shown in panel (c). Data are presented as the mean ± standard deviation from multiple fields of view across three independent experiments. (e) Hemichannel function assessed by DAPI uptake assay. DAPI reagent was added at the 0‐min time point, and an image was immediately captured as the initial baseline. A second image was captured at the 5‐min time point as the postuptake value. (f) Fluorescence intensity was calculated using ImageJ, and statistical analysis was performed using one‐way ANOVA.  ^∗^ *p* < 0.05,  ^∗∗^ *p* < 0.01,  ^∗∗∗^ *p* < 0.001,  ^∗∗∗∗^ *p* < 0.0001; ns, not statistically significant.

Moreover, functional experiments confirmed that these structural alterations resulted in significant channel dysfunction. The scrape‐loading dye transfer assay results (Figure 6c–d) showed that WT cells exhibited a clear ability to spread fluorescent dye to surrounding cells, whereas the dye diffusion area in the p.S42G mutant group was significantly reduced compared to the WT group (*p* < 0.0001), suggesting impaired gap junction‐mediated intercellular communication. Subsequently, hemichannel function was assessed via the DAPI uptake assay. The WT group demonstrated significant DAPI uptake capacity, whereas the mean fluorescence intensity of the p.S42G mutant group showed no significant difference (*ns*) compared to the negative control (NC) group and was significantly lower than that of the WT group (*p* < 0.01), indicating that this mutation leads to weakened Cx32 hemichannel function (Figure 6e–f).

## 4. Discussion

This study retrospectively analyzed the clinical manifestations and laboratory findings of eight patients with CMTX1. Through bioinformatics analysis combined with in vitro functional experiments, two novel *GJB1* variants (c.124A > G and c.130dupT), which have not been reported previously either domestically or internationally, were identified and characterized. The study elucidated two distinct molecular pathogenic mechanisms underlying CMTX1, “loss of expression” due to full‐length protein deficiency and “functional defect” resulting from channel constriction.

In the preliminary phase of the study, phenotypic analysis of the patients revealed that eight individuals carrying *GJB1* gene mutations exhibited clinical manifestations highly consistent with and characteristic of classic CMT. Additionally, rare central nervous system (CNS) involvement phenotypes, including nystagmus and abnormal signals in the splenium of the corpus callosum, were identified. These findings further support previous reports that *GJB1* mutations can lead to a phenotypic spectrum of combined central–peripheral involvement with transient CNS abnormalities [21]. Electrophysiological studies confirmed an intermediate pattern of nerve conduction impairment. Nerve biopsy results demonstrated features of chronic demyelination and axonal degeneration, consistent with prior research indicating that CMTX1 biopsy tissues typically exhibit normal myelin fiber density, predominantly thin myelination with a predominance of small fibers, rare onion bulb formation, and the presence of cluster structures [22]. In Proband 8, the pathology was characterized by slight axonal texture heterogeneity, vacuolation, and predominantly clustered small fibers with a small number of onion bulb‐like structures. Previous studies have suggested that the LOF Cx32^p.Val181Ala^ variant severely impairs the ability of Schwann cells to support the earliest stages of myelin fiber regeneration [23]. In contrast, the less severe Cx32^p.Glu102Gly^ variant allows Schwann cells to support normal early regeneration [24]. The variant in Proband 8 may belong to the category of mutations with less severe consequences. In some patients in our study, scattered distribution of CD68‐positive macrophages was also observed, potentially analogous to CSF‐1–activated macrophages mediating dedifferentiation of Cx32‐deficient Schwann cells, leading to demyelination [25]. Furthermore, genetic testing identified two novel variants not previously reported in the literature. Consequently, in‐depth investigations into their pathogenicity and underlying mechanisms were conducted.

Cx32 oligomerizes to form hexamers, known as connexons or hemichannels, which are transported to the cell membrane and dock with hemichannels from adjacent cells to form GJCs. [26, 27] GJCs provide direct cytoplasmic continuity for the exchange of ions and a broad range of signaling molecules, including cyclic nucleotides [28], inositol trisphosphate (IP3), [29] glucose [30], ATP [31], oligonucleotides [32], and small peptides [33], thereby supporting tissue homeostasis and coordinated responses to stimuli. Previous studies have demonstrated the critical roles of each structural domain of Cx32. The site formed by the N‐terminal gating helix binds phospholipids and can block the Cx32 GJCs [34], whereas the CL2 peptide exhibits high affinity for Ca2+/CaM [35]. When the channel is open, TM3 serves as the primary pore‐lining helix, with TM2 contributing to a lesser extent at the cytoplasmic end of the pore [36]. Subtle mutations can have severe consequences; for instance, the W3S and R22G mutant hemichannels exhibit a pore diameter reduction from 11 to 6 Å, which is demonstrated to impair the permeability of the channel to larger solutes such as ATP [37]. Therefore, alterations in protein structure and function following variant are a key focus of our investigation. For the *GJB1* c.124A > G variant, surface biotinylation assays and IF consistently showed that a portion of the mutant protein successfully reached the cell membrane, whereas another portion accumulated in the cytoplasm. The cytoplasmic accumulation is likely due to reduced hydrogen bonding in the junctional region, leading to local destabilization and triggering the endoplasmic reticulum quality control system, thereby retaining the protein intracellularly. This phenomenon has been similarly observed in mutations at other sites [38]. DAPI uptake assays revealed a significant reduction in hemichannel function compared to the wild type, untangling the negative impact of the mutation on channel function. In scrape‐loading dye transfer assays, the mutation markedly inhibited the intercellular diffusion of fluorescent dye, indicating impaired gap junction–mediated intercellular communication. Combined with molecular modeling results, we propose that this functional impairment is driven by a dual mechanism. First, pore diameter measurement in Chimera X revealed a physical constriction of 1.229 Å in the critical region of the pore. Although the overall hexameric symmetry was not disrupted, this constriction significantly increased the steric hindrance for the transmembrane transport of small molecular metabolites. Second, the increase in local positive electrostatic potential at the mutation site elevated the energy barrier for charged small molecules to traverse the pore. This combination of physical narrowing and electrostatic repulsion reduces the channel′s permeability to cations and essential metabolites, resulting in a “functional defect.” For the *GJB1* c.130dupT variant, mRNA expression was normal, but the full‐length Cx32 protein was undetectable by cellular WB and tissue immunohistochemistry, indicating a “loss of expression” of the full‐length Cx32 protein, analogous to *GJB1* gene knock out or large deletions. Previous studies in mice have shown that Cx32 knock out mice exhibit slowed motor nerve conduction velocity in the sciatic nerve as early as 3 months of age, which is earlier than in mice with knock‐in mutations (p.R75W and p.T55I). [39] Similarly, in this study, the age of onset for patients with the *GJB1* c.130dupT variant was earlier than that for patients with the *GJB1* c.124A > G variant.

We identified that both novel sites are located at the junction between TM1 and EC1, with only one amino acid residue separating the two mutation sites. Interestingly, however, their clinical manifestations are heterogeneous. Comparing the clinical presentations of the two patients with novel variants, it is evident that Proband 3 (c.130dupT) exhibited more severe symptoms and signs, including absent deep tendon reflexes, typical pes cavus, sensory impairment, and an earlier age of onset. In terms of neuropathological damage, Proband 3 demonstrated more profound tissue destruction, including a loss of large myelinated fibers, mild‐to‐moderate reduction in unmyelinated fibers, axonal shrinkage, degeneration, vacuolation, mild proliferation of interstitial fibroblasts, and epineurial edema. In contrast, Proband 5 (c.124A > G) presented with milder clinical symptoms, a later age of onset, and a slowly progressive course. Mechanistically, the latter mutation untangles a more complex pattern. Although a portion of the protein is successfully trafficked to the plasma membrane, the actual permeation function of the channel is compromised due to localized physical constriction in the TM1‐ECL1 region and an elevated electrostatic potential barrier. This “present but impaired” functional state explains the progressive nature of the clinical manifestations.

It is noteworthy that the Proband 5 exhibited features of CNS involvement, with MRI revealing white matter degeneration. Transient CNS symptoms in CMTX1 are often triggered by metabolic stressors, including high‐altitude exposure (12.8%) [40], fever, or hyperventilation. [41] The proband reported that during the first hospitalization, he had gone hiking and experienced weakness in both legs upon reaching the mountain summit, requiring assistance to descend. Studies have also indicated that in the CNS, Cx32 often forms heterotypic coupling with Cx47, and the p.S42G mutation may interfere with this heterotypic coupling between Cx32 and Cx47 in oligodendrocytes. This finding suggests that when evaluating novel GJB1 mutations, the dual impact on gap junction function in both the central and peripheral nervous systems should be considered concurrently.

In summary, this study expands the pathogenic variant spectrum of the *GJB1* gene and untangles the molecular pathological mechanisms of CMTX1 from the dual perspectives of “expression” and “function,” providing a profound experimental basis for the precise diagnosis and subsequent intervention research of this disease. However, some limitations remain, such as the absence of patch‐clamp electrophysiology to measure channel currents and the lack of animal models to simulate the long‐term pathological process. Future work will focus on continuous improvement and in‐depth exploration of the underlying mechanisms.

## Funding

This study was supported by the Science and Technology Projects in Guangzhou, 2024B03J1257.

## Conflicts of Interest

The authors declare no conflicts of interest.

## Supporting information


**Supporting Information** Additional supporting information can be found online in the Supporting Information section. Table S1: Clinical characteristics of eight probands with Charcot–Marie–Tooth disease. Table S2: Neurophysiological Findings. Table S3: Nerve biopsy pathology of eight patients. Table S4: Mutation analysis of GJB1 in eight patients.

## Data Availability

The data that support the findings of this study are available on request from the corresponding author. The data are not publicly available due to privacy or ethical restrictions.
