# Supplementary material for: Pathogenic Mechanisms of Two Novel Variants in the GJB1 Gene
Source: Hum Mutat. 2026 Oct 9;2026:7388252. doi: 10.1155/humu/7388252 (PMC13651715; doi:10.1155/humu/7388252)
Supplement: Supplementary file 1 [file HUMU-2026-7388252-s001.docx]

**Sup. Table 1 Clinical characteristics of eight probands with Charcot-Marie-Tooth disease.**

| Family/Proband | 1 | 2 | 3 | 4 | 5 | 6 | 7 | 8 |
| --- | --- | --- | --- | --- | --- | --- | --- | --- |
| Age（years） | 36 | 18 | 28 | 16 | 40 | 22 | 38 | 23 |
| Sex | male | male | male | male | male | male | male | male |
| Age at onset（years） | 25 | 15 | 23 | 6 | 36 | 13 | 33 | 16 |
| Initial symptoms | Lower limb muscle atrophy and weakness | Frequent tripping while running | Lower limb muscle weakness | Lower limb muscle atrophy | Lower limb muscle weakness | Lower limb muscle weakness | Foot drop | Weakness of all four limbs |
| Atrophy UL/LL | N/3+ | 1+/3+ | 1+/3+ | N/2+ | N/2+ | 1+/3+ | N/2+ | 2+/2+ |
| Deep reflexes UL/LL | +/+ | +/+ | -/- | ++/++ | +/+ | +/+ | +/- | -/- |
| Pes cavus | No | No | Yes | Yes | No | Yes | No | Yes |
| Sensation loss | No | No | hypalgesia | Decreased facial sensation | + | hypalgesia | No | No |
| Nystagmus | No | No | No | Yes | No | No | No | No |
| Brain MRI | normal | normal | normal | Abnormal^1^ | Abnormal^2^ | N/A | Abnormal^3^ | Abnormal |

Note:^1^Abnormal signal intensity in the splenium of the corpus callosum. ^2^Mild leukoencephalopathy (white matter degeneration) in the bilateral supratentorial hemispheres; sinusitis involving the left frontal sinus, sphenoid sinus, bilateral ethmoid sinuses, and right maxillary sinus. ^3^The left lateral ventricle is enlarged compared to the contralateral side. Abbreviations and definition: UL, Upper limbs; LL, Lower limbs; N, Normal; 1+, Mild; 2+, Moderate; 3+, Severe; -, Disappeared; +, Decreased; ++, Normal; +++, Increased; N/A, Not Applicable.

**Sup. Table 2 Neurophysiological Findings**

| **Value** | | **N** | **Mean** | **SD** | **Median** | **Range** | **Abnormal** |
| --- | --- | --- | --- | --- | --- | --- | --- |
| Age at examination，y | | 6 | 35.5 | 8.5 | 36.5 | 22-47 | - |
| **Sensory nerve action poteintials** | | | | | | | |
| Median | Amp, μV(≥8) | 6 | 5.49 | 3.68 | 4.51 | 1.82-12 | 5 |
|  | CV, m/s (≥50) | 6 | 36.57 | 3.597 | 34.65 | 33-42.9 | 6 |
| Ulnar | Amp, μV(≥5) | 4 | 5.58 | 3.5 | 5.45 | 1.41-10 | 2 |
|  | CV, m/s (≥50) | 4 | 36.775 | 4.367 | 34.8 | 33.3-44.2 | 4 |
| Sural | Amp, μV(≥20) | 4 | 5.29 | 2.75 | 5.615 | 1.94-8 | 4 |
|  | CV, m/s (≥50) | 4 | 34.4 | 2.08 | 33.3 | 33-38 | 4 |
| **Compound muscle action potentials** | | | | | | | |
| Median SE on APB | Amp, μV(≥5) | 6 | 4.69 | 1.676 | 5.3 | 1.16-6.1 | 2 |
|  | CV, m/s (≥50) | 5 | 32.88 | 7.79 | 33.3 | 21-45.1 | 5 |
| Ulnar SE on ADM | Amp，μV(≥8) | 6 | 7.11 | 2.59 | 7.54 | 2.5-10.3 | 3 |
|  | CV, m/s (≥50) | 6 | 40.25 | 6.75 | 37.55 | 32-53 | 5 |
| C peroneal SE on EDB | Amp, μV(≥2.5) | 5 | 2.026 | 1.943 | 0.9 | 0.16-4.32 | 3 |
|  | CV, m/s (≥40) | 3 | 27.5 | 11.23 | 28 | 13.5-41 | 2 |
| P titial SE on AH | Amp, μV(≥4) | 5 | 0.708 | 0.465 | 0.56 | 0.1-1.4 | 5 |
|  | CV, m/s (≥40) | 1 | 26 | 0 | 26 | 26-26 | 1 |

Note: N, Number of patients; ADM, Abductor Digiti Minimi; AH, Abductor Hallucis.

Amp, Amplitude; APB, Abductor Pollicis Brevis; CV, Conduction Velocity; EDB, Extensor Digitorum Brevis; P, Posterior; SE, Surface Electrode.

S**up. Table 3 Nerve biopsy pathology of eight patients.**

| **Proband** | **P1** | **P2** | **P3** | **P4** | **P5** | **P6** | **P7** | **P8** |
| --- | --- | --- | --- | --- | --- | --- | --- | --- |
| **Epineurium** |  |  |  |  |  |  |  |  |
| hyperplasia | 0 | 1 | 0 | 0 | 1 | 1 | 1 | 1 |
| edema | 0 | 0 | 1 | 0 | 0 | 0 | 0 | 0 |
| inflammatory cell infiltration | 1 | 1 | 1 | 0 | 1 | 0 | 0 | 1 |
| **Perineurial** |  |  |  |  |  |  |  |  |
| thickening | 0 | 0 | 0 | 0 | 0 | 1 | 0 | 0 |
| edema | 1 | 0 | 1 | 1 | 1 | 0 | 0 | 1 |
| **Blood vessel** |  |  |  |  |  |  |  |  |
| thickening | 1 | 0 | 0 | 0 | 0 | 0 | 0 | 0 |
| number （CD34） | 1 | 0 | 0 | 0 | 1 | 0 | 0 | 0 |
| **Nerve fibers** |  |  |  |  |  |  |  |  |
| large myelinated fibers | 4 | 3 | 5 | 1 | 1 | 1 | 4 | 4 |
| small myelinated fibers | 2 | 3 | 0 | 1 | 1 | 1 | 6 | 2 |
| unmyelinated fibers（NF） | 2 | 1 | 2 | 2 | 1 | 1 | 1 | 2 |
| **Myelin** |  |  |  |  |  |  |  |  |
| renaut bodies | 0 | 0 | 0 | 0 | 1 | 0 | 0 | 0 |
| thinly myelinated fibers | 1 | 1 | 1 | 0 | 2 | 3 | 0 | 2 |
| small rosette-like | 0 | 2 | 1 | 0 | 2 | 1 | 0 | 0 |
| lamellation | 1 | 1 | 0 | 1 | 4 | 1 | 0 | 2 |
| myelin globule | 0 | 0 | 0 | 0 | 2 | 0 | 0 | 0 |
| **Axons** |  |  |  |  |  |  |  |  |
| axonal vacuoles | 2 | 1 | 1 | 1 | 3 | 1 | 0 | 2 |
| small fibers clustering | 2 | 0 | 1 | 0 | 0 | 3 | 0 | 0 |
| **IHC** |  |  |  |  |  |  |  |  |
| CD4 | 1 | 1 | 0 | 3 | 3 | 0 | 0 | 0 |
| CD8 | 1 | 1 | 1 | 1 | 3 | 1 | 1 | 1 |
| CD68 | 3 | 1 | 3 | 3 | 2 | 2 | 2 | 3 |
| CD79α | 0 | 0 | 0 | 1 | 1 | 1 | 1 | 0 |

Note：In the table, 5/8 epineurium hyperplasia, 5/8 epineurium inflammatory cell infiltration, only 1/8 perineurial thickening, 5/8 perineural edema, only 1/8 blood vessel thickening, 2/8 blood vessel number increase. All showed a reduction in large myelinated fibers, with 5/8 demonstrating a moderate or greater degree of decrease. Small myelinated fibers exhibited a mild to moderate reduction, with one participant even showing an increase. Unmyelinated fibers showed a mild （4/8） or mild-to-moderate （4/8）reduction. Renaut bodies were identified in one myelin sheath. 6/8 had thinly myelinated fibers, 4/8 had small rosette-like myelin, 6/8 had lamellation. Myelin globule were identified in one myelin sheath. 7/8 had axonal vacuoles, 3/8 had small fibers clustering. Inflammatory cell infiltration in all participants, with CD8+ and CD68+ cells present at varying levels. CD8+ cells were observed in seven participants, while CD68+ cells were found in four. As for the definitions of each item, P1-8 refers to proband 1–8. Epineurium, Perineurial, and Blood vessel are denoted using 0-No and 1-Yes. For Nerve fibers, a 0-5 scale is used to indicate the degree of decrease: 0-No, 1-mild, 2-mild to moderate, 3-moderate, 4-moderate to severe, and 5-severe, while 6 is used to indicate an increase. The definitions of positive manifestations in myelin, axons, and IHC are as follows: Under a 200x field of view, Occasional: The number of positive cells per field of view is ≤ 1, with sparse distribution. Scattered: The number of positive cells per field of view is 2-5, showing no aggregation tendency, randomly distributed in the tissue, and not forming small clusters or foci. Small amount: The number of positive cells per field of view is 6-20, which can be scattered or form small clusters (aggregation of ≤ 3 cells), but do not occupy the main area of the field of view. A large number: The number of positive cells per field of view is ＞20, or positive cells account for more than 30% of the total number of cells in the area, which can be distributed diffusely or in large patches. IHC: Immunohistochemistry.

**Sup. Table 4 Mutation analysis of GJB1 in eight patients.**

| proband | family | Variant position | cDNA change | Protein change | Novelty |
| --- | --- | --- | --- | --- | --- |
| 1 | F1 II-5 | Exon | c.238C>T | p.Gln80* | Reported |
| 2 | F2 III-6 | Intron | c.-459C>T | p.(=) | Reported |
| 3 | F3 II-1 | Exon | c.130dupT | p.(Trp44LeufsTer3) | Novel |
| 4 | F4 II-1 | Exon | c.319C>T | p.(Arg107Trp) | Reported |
| 5 | F5 II-3 | Exon | c.124A>G | p.(Ser42Gly) | Novel |
| 6 | F6 III-2 | Exon | c.548G>A | p.(Arg183His) | Reported |
| 7 | F7 II-9 | Exon | c.283G>A | p.(Val95Met) | Reported |
| 8 | F8 III-2 | Intron | c.-103C>T | p.(=) | Reported |

Note: F1-8 refers to family 1–8.
